# Interleukin-32 as a biomarker in rheumatic diseases: A narrative review

**DOI:** 10.3389/fimmu.2023.1140373

**Published:** 2023-02-15

**Authors:** Oh Chan Kwon, Min-Chan Park, Yong-Gil Kim

**Affiliations:** ^1^ Division of Rheumatology, Department of Internal Medicine, Yonsei University College of Medicine, Seoul, Republic of Korea; ^2^ Division of Rheumatology, Department of Internal Medicine, University of Ulsan, College of Medicine, Asan Medical Center, Seoul, Republic of Korea; ^3^ Convergence Medicine Research Center, Asan Institution for Life Science, Asan Medical Center, Seoul, Republic of Korea

**Keywords:** IL-32, biomarker, rheumatic disease, inflammatory arthritis, connective tissue disease

## Abstract

Interleukin-32 (IL-32) is an important cytokine involved in the innate and adaptive immune responses. The role of IL-32 has been studied in the context of various diseases. A growing body of research has investigated the role of IL-32 in rheumatic diseases including inflammatory arthritides (rheumatoid arthritis, ankylosing spondylitis, and psoriatic arthritis) and connective tissue diseases (systemic lupus erythematosus, systemic sclerosis, granulomatosis and polyangiitis, and giant cell arteritis). IL-32 has been shown to play different roles according to the type of rheumatic diseases. Hence, the putative role of IL-32 as a biomarker is also different in each rheumatic disease: IL-32 could serve as a biomarker for disease activity in some diseases, whereas in other diseases it could be a biomarker for certain disease manifestations. In this narrative review, we summarize the associations between IL-32 and various rheumatic diseases and discuss the putative role of IL-32 as a biomarker in each disease.

## Introduction

Interleukin-32 (IL-32), formerly known as the Natural Killer Cell Transcript 4 (NK4), is a cytokine that plays an important role in both innate and adaptive immune responses (1–4). In various inflammatory stimuli, IL-32 is produced by various cells, including monocytes, T cells, NK cells, fibroblasts, epithelial cells, and endothelial cells (2, 5, 6). IL-32, in turn, stimulates monocytes to differentiate into macrophage or dendritic cells and induces pro-inflammatory cytokines including tumor necrosis factor (TNF)-α, IL-1β, IL-6, and IL-8 through nuclear factor-kappa B (NF-κB) and p38 mitogen-activated protein kinase inflammatory signal pathway (2, 7). Since its identification in 1992, IL-32 has been studied in a wide range of diseases including chronic inflammatory diseases, autoimmune diseases, infection, and cancer (8–11). Rheumatic diseases are one of the disease groups that the role of IL-32 has been extensively studied on (12–18).

The *IL-32* gene is found in higher mammals but not in rodents (2). The gene is located in chromosome 16p13.3 and has eight exons (2). The first exon does not translate into amino acids (3). Based on different alternative splicing sites, IL-32 has several isoforms: IL-32α, IL-32β, IL-32γ, IL-32δ, IL-32ϵ, IL-32ζ, IL-32η, IL-32θ, and IL-32small (IL-32sm) (19). The biological activity of IL-32 varies according to the isoforms with IL-32γ and IL-32θ having the most potent biological activity (7, 20, 21). Of the isoforms, IL-32γ has the longest amino acid sequence and is the isoform that is most studied (20, 22). All isoforms have a tripeptide RGD motif, which helps cell adhesion and movement (23). Through these motifs, the secreted forms of IL-32 may bind to integrin, suggesting integrin as a possible receptor for IL-32 (24). However, specific receptors for IL-32 are yet to be identified.

Biomarkers are defined as a marker that can be objectively measured and evaluated as an indicator of normal biological processes, pathogenic processes, or pharmacologic responses to a therapeutic intervention (25). In the field of rheumatic diseases, biomarkers are increasingly used for early diagnosis of the disease, accurate assessment of disease activity, detection of certain disease manifestations, and prediction of treatment response and prognosis (26). A growing body of evidence suggests that IL-32 could be used as a possible biomarker in various rheumatic diseases. In this narrative review, we summarize the associations between IL-32 and various rheumatic diseases, and discuss the potential role of IL-32 as a biomarker in each disease.

## Inflammatory arthritides

### Rheumatoid arthritis

Rheumatoid arthritis (RA) is a chronic autoimmune disease characterized by inflammatory synovitis, which leads to progressive joint damage (27). The inflammatory milieu of the synovial tissue is mediated by pro-inflammatory cytokines such as TNF-α, IL-1β, and IL-6 (28). IL-32, as a pro-inflammatory cytokine, has also been implicated in the pathogenesis of synovitis in RA (29). In synovial tissue from patients with RA, IL-32 was highly expressed, whereas in that from patients with osteoarthritis (OA), expression of IL-32 was not observed (29). In addition, the level of IL-32 staining in synovial tissue from patients with RA correlated with erythrocyte sedimentation rate (ESR), indices of synovial inflammation, and synovial presence of TNF-α, IL-1β, and IL-18 (29). In a study comparing gene expression profiles of *in vitro* cultured fibroblast-like synoviocytes (FLS) from patients with RA and OA, *IL-32* was the most prominent gene that was differentially expressed between patients with RA and OA, with higher expression in patients with RA (30). These findings together indicate that IL-32 is an important cytokine involved in the pathogenesis of synovial inflammation in RA (29, 30).

The suggested mechanism of IL-32 for inducing inflammatory synovitis in RA is induction of other pro-inflammatory cytokines that are involved in the development of RA (28). When human IL-32γ was injected into knee joints of C57/BL6 (wild-type [WT]) mice, joint swelling, infiltration of inflammatory cells, and cartilage damage were observed, whereas in TNF-α deficient mice, the injection of human IL-32γ into the knee joints did not result in joint swelling, and the inflammatory cell influx was markedly reduced (29). In another study using a collagen-induced arthritis model, transfer of IL-32β-producing CD4+ T cells aggravated arthritis, which was attenuated by TNF-α blockade (6). These findings suggest that the pathogenic role of IL-32 in RA is at least in part, dependent on TNF-α. Conversely, TNF-α also induces expression of IL-32 in synovial fibroblasts, monocyte-derived dendritic cells, and T cells (6). In response to TNF-α, FLS from patients with RA produce IL-32 in a dose-dependent manner, indicating that inflammatory cascade in the synovial tissue can be magnified *via* IL-32 activity through an autocrine loop (31). In addition, IL-32γ induces the maturation of dendritic cells and production of IL-12, leading to enhanced Th1 response, which may aggravate inflammation in RA (32).

In addition to its role in inducing inflammation in synovial tissue, IL-32 also plays a role in bone resorption in RA (13). IL-32α induces differentiation of osteoclast precursors into multinucleated osteoclasts, partially independent of the receptor activator of nuclear factor kappa B (RANK)/RANK ligand (RANKL) pathway (33). However, IL-32α is not capable of activating the multinucleated osteoclasts into bone-resorbing osteoclasts (33). In contrast, IL-32γ, which is the isoform with a stronger biological activity, potently stimulates both the differentiation of osteoclast precursor into multinucleated osteoclast and the activation of multinucleated osteoclast into bone-resorbing osteoclasts, with synergistic effect with RANKL (34). Furthermore, IL-32γ increases the expression of RANKL but decreases the expression of osteoprotegerin in FLS from patients with RA, which is a favorable condition for osteoclastogenesis, leading to bone resorption (34).

Taken together, IL-32 could be a putative biomarker for assessing inflammatory burden (i.e., disease activity) and the isoform IL-32γ could be a biomarker for bone resorption in RA (**Table 1**).

**Table 1:** Putative role of IL-32 as a biomarker in inflammatory arthritides.

Disease	Role in disease/disease association	Putative role as a biomarker
RA	Induces synovitis Induces osteoclast differentiation and activation leading to bone resorptive phenotype	Biomarker for disease activity Biomarker for bone resorption
AS	Enhances osteoblast differentiation by suppressing DKK-1 leading to abnormal new bone formation Involved in intestinal inflammation in AS with a protective role	Biomarker for abnormal new bone formation Biomarker for detecting intestinal inflammation
PsA	Serum levels higher in PsA than in controls, accompanied by higher levels of activated NF-κB Serum levels higher in PsA than in psoriasis	Biomarker for disease activity Biomarker for development of PsA in patients with psoriasis

IL, interleukin; RA, rheumatoid arthritis; AS, ankylosing spondylitis; DKK-1, Dickkopf-1; PsA, psoriatic arthritis; NF-κB, nuclear transcription factor-κB.

### Ankylosing spondylitis

Ankylosing spondylitis (AS) is a chronic inflammatory arthritis that mainly affects the axial skeleton with a characteristic bone phenotype of abnormal new bone formation (35). The pathogenic role of IL-32γ in bone formation in AS has been suggested (36, 37). Compared with patients with OA and even those with RA, patients with AS have higher levels of IL-32γ in synovial fluid and higher expression of IL-32 in synovial tissue (36). When osteoblast precursors from WT (C57/BL6) mice were stimulated with IL-32γ, osteoblast differentiation was potently enhanced (36). Furthermore, IL-32γ transgenic mice had higher rates of osteoblast differentiation than the WT mice (36). Mechanistically, IL-32γ reduced the expression of Dickkopf-1 (DKK-1), an inhibitor of Wnt/β-catenin signaling pathway in osteoblast precursors (36). The reduced expression of DKK-1 was mediated by upregulation of miR-29a in osteoblast precursors (37). These data indicate that IL-32γ is involved in abnormal new bone formation in AS by suppressing DKK-1 expression, resulting in enhanced osteoblast differentiation.

IL-32 has also been implicated in intestinal inflammation in AS (38). Ileal tissue from patients with AS who had chronic intestinal inflammation revealed higher expression of IL-32 than that from patients with AS without chronic intestinal inflammation, and healthy controls (38). Functionally, IL-32 stimulated IL-10 production in human intestinal epithelial cell line, suggesting that IL-32 is involved in intestinal inflammation in AS with a protective role (38).

Collectively, IL-32γ could be a biomarker for abnormal new bone formation and IL-32 could be a biomarker for detecting intestinal inflammation in AS (**Table 1**).

### Psoriatic arthritis

Psoriatic arthritis (PsA) is a chronic inflammatory arthritis that affects approximately 30% of patients with psoriasis (39). A study using plasma samples has shown that the levels of IL-32 were higher in the plasma from patients with PsA than that from healthy controls (14). Moreover, levels of activated NF-κB were higher in peripheral blood mononuclear cells (PBMCs) from patients with PsA than that from healthy controls: although direct evidence of association between IL-32 and activation of NF-κB has not been provided, the study suggested a possible role of IL-32 in inflammatory process in PsA (14).

Psoriasis precedes PsA in a majority of the cases of PsA (39). Interestingly, the levels of IL-32 in the plasma and *IL-32* mRNA in the PBMCs from patients with psoriasis are already higher than those from healthy controls (14, 40). In comparison between patients with PsA and those with psoriasis, the levels of IL-32 in plasma from patients with PsA were higher than that from patients with psoriasis (14). These findings suggest that levels of IL-32 in the plasma from patients with psoriasis increase before PsA develops, which further increase on the development of PsA. Therefore, IL-32 could be a biomarker for assessing disease activity of PsA and for detecting development of PsA in patients with psoriasis (**Table 1**).

## Connective tissue diseases

### Systemic lupus erythematosus

Systemic lupus erythematosus (SLE) is a systemic autoimmune disease that can cause inflammation and damage in virtually all organs throughout the body (41). In a study from China comparing serum levels of various cytokines between patients with SLE and healthy controls, there was no significant difference in the serum levels of IL-32 (42). Another study, also from China, reported lower plasma levels of IL-32 in patients with SLE than in healthy controls (43). The authors speculated that the levels of IL-32 could have been measured low in patients with SLE because of the treatment they received (42, 43). There is evidence suggesting that IL-32 is involved in the pathogenesis of SLE, in particular, lupus nephritis (LN) (15, 44). In a study that measured serum levels of IL-32γ in patients with SLE and healthy controls, serum levels of IL-32γ were detectable in 18.8% of patients with LN, whereas the levels were not detectable in patients with SLE without history of LN and in healthy controls, suggesting a possible pathogenic role of IL-32γ in LN (44). Similarly, in a study with larger sample size, serum levels of IL-32γ were higher in patients with LN than in patients with SLE without LN and healthy controls (15). The serum level of IL-32γ positively correlated with renal component of SLE disease activity index, histologic activity index, and histologic chronicity index of LN (15). Moreover, the expression of IL-32 in renal tissue was higher in patients with LN than in healthy controls (15). Taken together, IL-32γ could be a biomarker for detecting development of LN in patients with SLE and for assessing disease activity of LN (**Table 2**).

**Table 2:** Putative role of IL-32 as a biomarker in connective tissue diseases.

Disease	Role in disease/disease association	Putative role as a biomarker
SLE	Serum levels higher in LN than in SLE without LN, and controls Serum levels correlate with renal component of SLEDAI, and histologic AI and CI	Biomarker for development of LN in patients with SLE Biomarker for disease activity of LN
SSc	Serum levels higher is SSc with PAH than in SSc without PAH, and idiopathic PAH IL-32+ cells in skin tissue correlate with mRSS	Biomarker for development of PAH in patients with SSc Biomarker for predicting prognosis
GPA	Serum levels and mRNA levels in leukocyte higher in GPA than in controls Serum levels associated with disease activity and decrease upon treatment	Biomarker for diagnosis Biomarker for monitoring treatment response
GCA	Arterial expression levels of protein and mRNA higher in GCA than in control Overexpression of IL-32 accompanied by Th1 cytokines Th1 lymphocytes expanded in GCA, producing higher amounts of IL-32 than controls	Biomarker for disease activity

IL, interleukin; SLE, systemic lupus erythematosus; LN, lupus nephritis, SLEDAI, systemic lupus erythematosus disease activity index; AI, activity index; CI, chronicity index; SSc, systemic sclerosis; PAH, pulmonary arterial hypertension; mRSS, modified Rodnan skin score; GPA, granulomatosis with polyangiitis; GCA, giant cell arteritis.

### Systemic sclerosis

Systemic sclerosis (SSc) is an autoimmune disease characterized by microvascular damage and progressive fibrosis of the skin and internal organs (45). IL-32 has been tested as a biomarker for detection of pulmonary arterial hypertension (PAH) in patients with SSc (16). Compared with patients with SSc without PAH and those with idiopathic PAH, patients with SSc with PAH had higher levels of serum IL-32, suggesting IL-32 as a promising biomarker for detecting PAH in patients with SSc (16). Moreover, the serum levels of IL-32 correlated with mean pulmonary arterial pressure and systolic pulmonary arterial pressure indicating that IL-32 could be used as a new screening tool for PAH in patients with SSc (16). The number of IL-32^+^ cells was higher in skin tissue derived from patients with SSc with PAH than that from patients with SSc without PAH (16). In addition, the number of IL-32^+^ cells in the skin tissue correlated with the modified Rodnan skin score (mRSS) (16). Given that mRSS is considered as a surrogate outcome measure for severity and mortality in patients with SSc (46, 47), IL-32^+^ cells in the skin tissue could have prognostic value. Therefore, IL-32 could be a biomarker for detecting development of PAH and predicting prognosis in SSc (**Table 2**).

### Granulomatosis with polyangiitis

Granulomatosis with polyangiitis (GPA) is a systemic vasculitis characterized by necrotizing granulomatous inflammation involving the respiratory tract and necrotizing vasculitis affecting small vessels (48). Proteinase 3 (PR3) is a major autoantigen targeted by anti-neutrophil cytoplasmic antibodies in GPA (49). PR3 is also known to specifically bind and activate IL-32 (50). On this basis, a study has evaluated the role of IL-32 in GPA (17). The serum levels of IL-32 were higher in patients with GPA than in healthy controls, attributable to the higher levels *IL-32* mRNA in leukocytes from patients with GPA than that from healthy controls (50). The serum levels of IL-32 were associated with disease activity as assessed by Birmingham vasculitis activity score, which decreased in response to treatment (50). These data suggest that IL-32 could be a biomarker for diagnosing GPA and monitoring treatment response.

### Giant cell arteritis

Giant cell arteritis (GCA) is a systemic vasculitis that primarily affects large- and medium-sized vessels (48). The levels of IL-32 and *IL-32* mRNA were higher in arterial biopsy specimens from patients with GCA than that from healthy controls (18). Moreover, the expression level of IL-32 correlated with the number of inflammatory parameters (fever, weight loss, ESR ≥85 mm/h, and hemoglobin <11.0 g/dL) (18). In the inflamed arteries from patients with GCA, the overexpression of IL-32 was accompanied by overexpression of interferon-γ and IL-27p28, which are Th1 cytokines (18). Th1 lymphocytes were expanded among PBMCs from patients with GCA and produced higher amounts of IL-32 compared with that in healthy controls (18). These suggest that IL-32 has an important role in mediating arterial inflammation in GCA. Therefore, IL-32 could be a biomarker for disease activity of GCA (**Table 2**).

## Conclusion

A number of studies have shown varying roles of IL-32 depending on the type of rheumatic diseases, indicating its different role as a putative biomarker in each disease. Although this progress is encouraging and could help clinicians in their clinical practice, we are still far from completely understanding the exact mechanism underlying the associations between IL-32 and each rheumatic disease. Considering the lack of recognized specific IL-32 receptor, identification of the IL-32 receptor could be the first step in elucidating the mechanism. Moreover, majority of the previous studies did not specify the isoform of IL-32. As different isoforms exert different biological activity, it is important to specify the isoform of IL-32 in future studies. Finally, although a variety of rheumatic diseases have been studied, data on connective tissue diseases are still relatively scarce. Further validation studies assessing IL-32 as a biomarker in the diseases summarized here are definitely needed.

## Author contributions

Y-GK was responsible for the concept of the manuscript. OCK drafted the manuscript. M-CP and Y-GK revised the manuscript. All authors contributed to the article and approved the submitted version.
